# Supplementary material for: NADES Compounds Identified in Hypoxis hemerocallidea Corms during Dormancy
Source: Plants (Basel). 2022 Sep 13;11(18):2387. doi: 10.3390/plants11182387 (PMC9503605; doi:10.3390/plants11182387)
Supplement: Supplementary file 1 [file plants-11-02387-s001.zip › plants-1878761-supplementary.pdf]

NADES Compounds Identified in *Hypoxis hemerocallidea* Corms during Dormancy

Motiki M. Mofokeng[1,2*], Gerhard Prinsloo[3], Hintsa T. Araya[1,*], Stephen O. Amoo[1,4,*] Christian P. du Plooy[1] and P.W. Mashela[2]

[1] Agricultural Research Council – Vegetable, Industrial and Medicinal Plants (ARC-VIMP), Private Bag X293, Pretoria, South Africa, 0001
[2] Green Technologies Research Centre, University of Limpopo, Private Bag X1106, Sovenga, South Africa, 0727
[3] Department of Agriculture and Animal Health, University of South Africa, Private Bag X6, Johannesburg, South Africa, 1710
[4] Department of Botany and Plant Biotechnology, University of Johannesburg, P.O. Box 524, Auckland Park, Johannesburg, South Africa, 2006
* Correspondence: MofokengM@arc.agric.za, MM; ArayaH@arc.agric.za, HT; AmooS@arc.agric.za, SO; Tel.: +27 12 808 8000

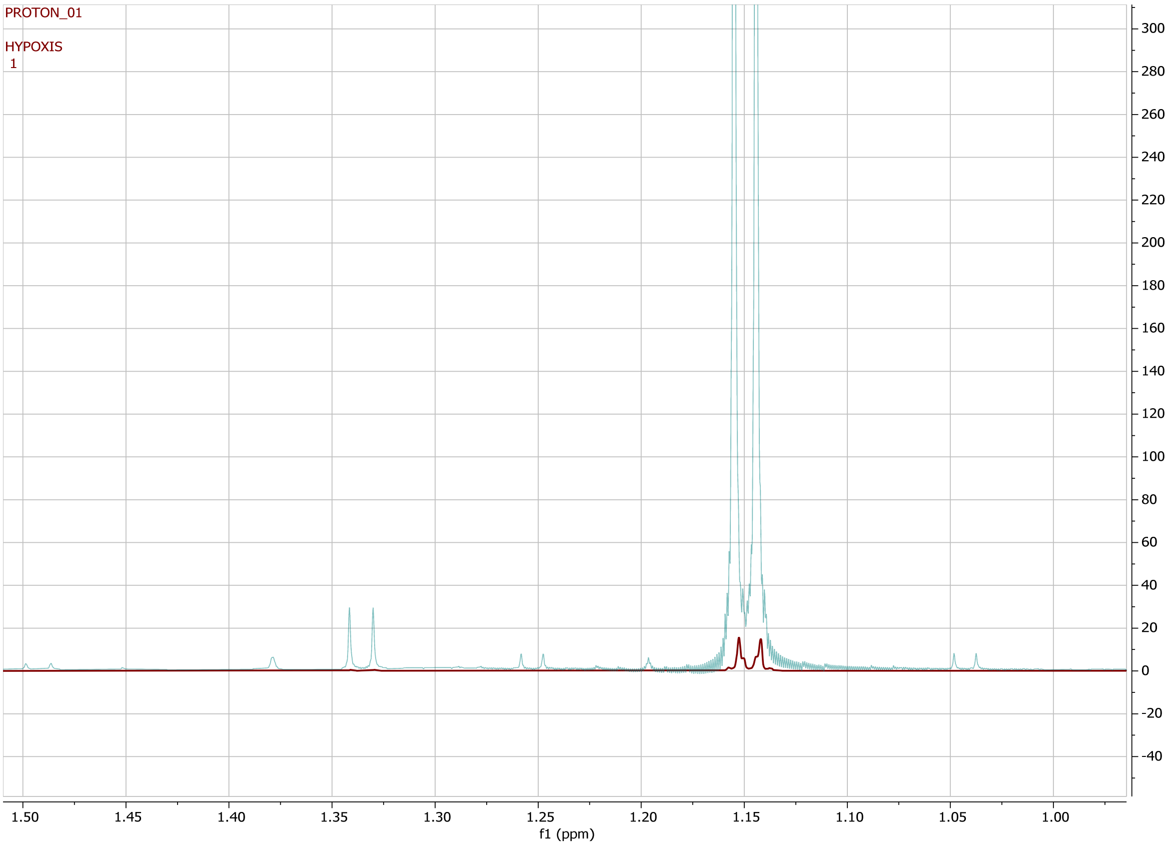

Figure S1. Spiking of the leachate (red) with the standard of propylene glycol. The two peaks align at 1.14 and 1.16 ppm with identical peak shapes.

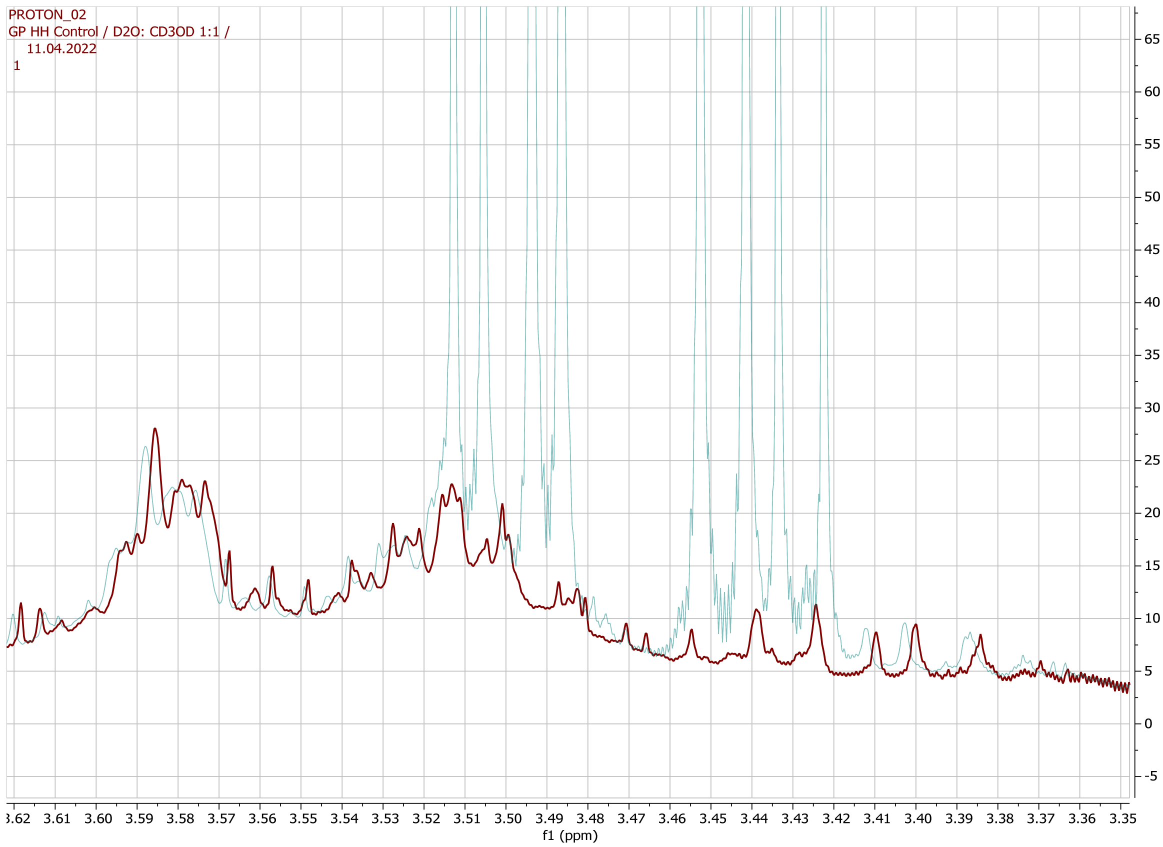

Figure S2. Spiking of the leachate (red) sample with propylene glycol with the peaks aligned at 4.24, 3.44, 43.46, 3.49, 3.50 and 3.53 ppm.

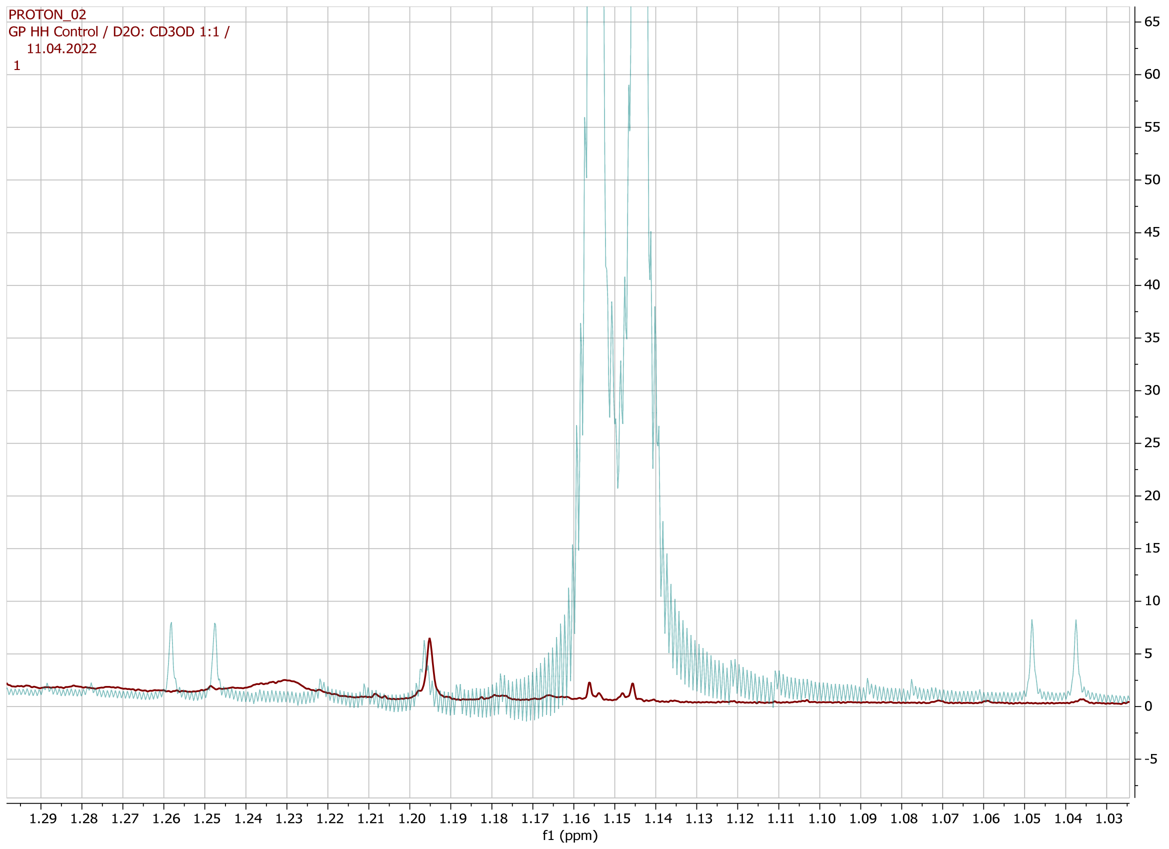

Figure S3. Spiking of the leachate (red) sample with propylene glycol showing alignment of the peaks at 1.15 and 1.16 ppm.

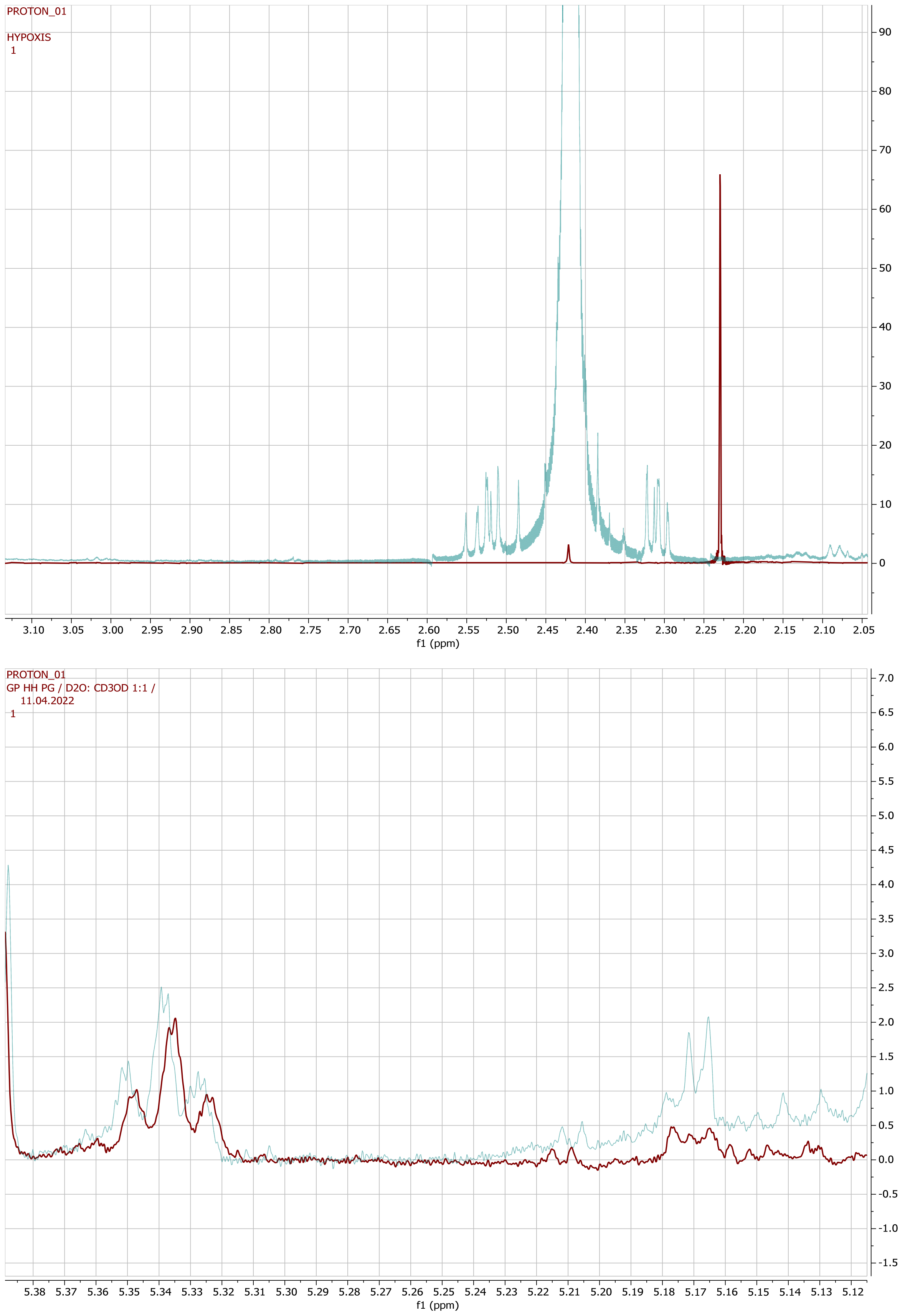

Figure S4. Spiking of the leachate sample with the pure standard of succinate. The peaks align at 2.42 and 5.17 ppm.

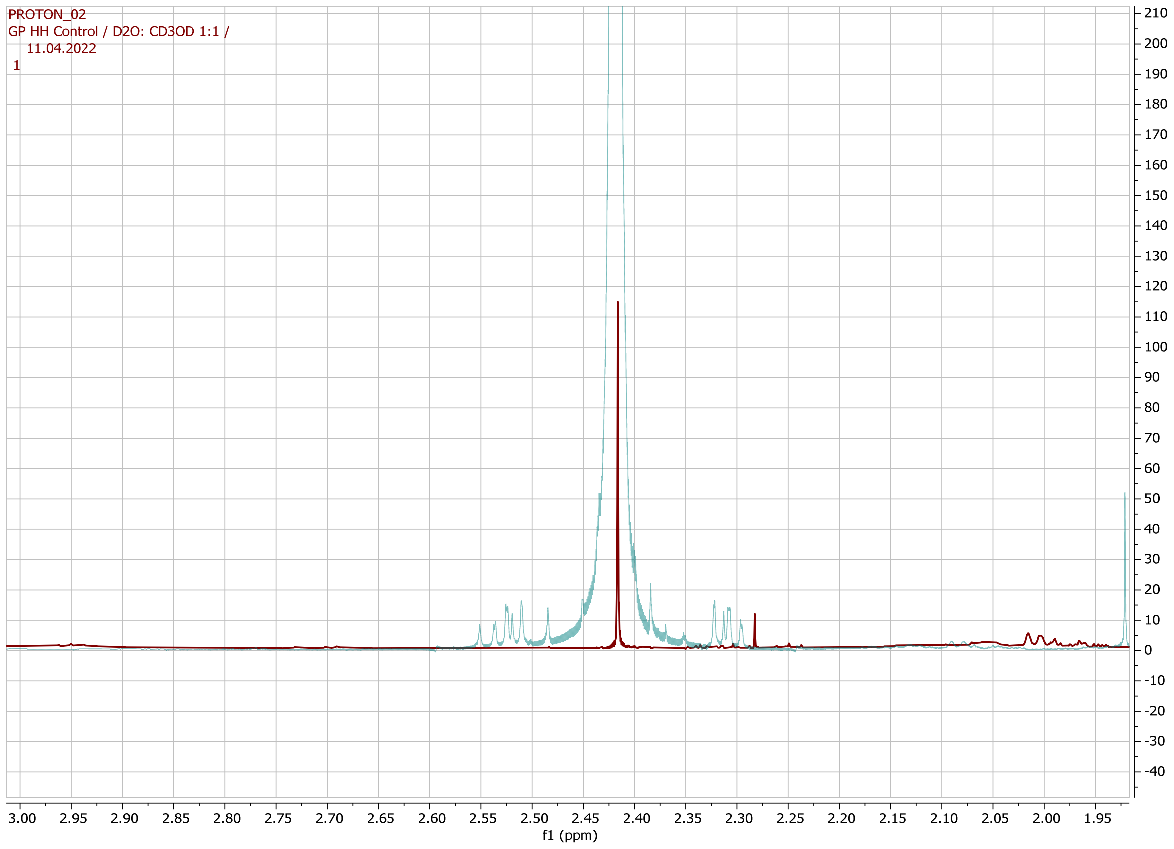

Figure S5. Spiking of the leachate sample (red) with the pure standard of succinate showing alignment of the peak at 2.43 ppm.

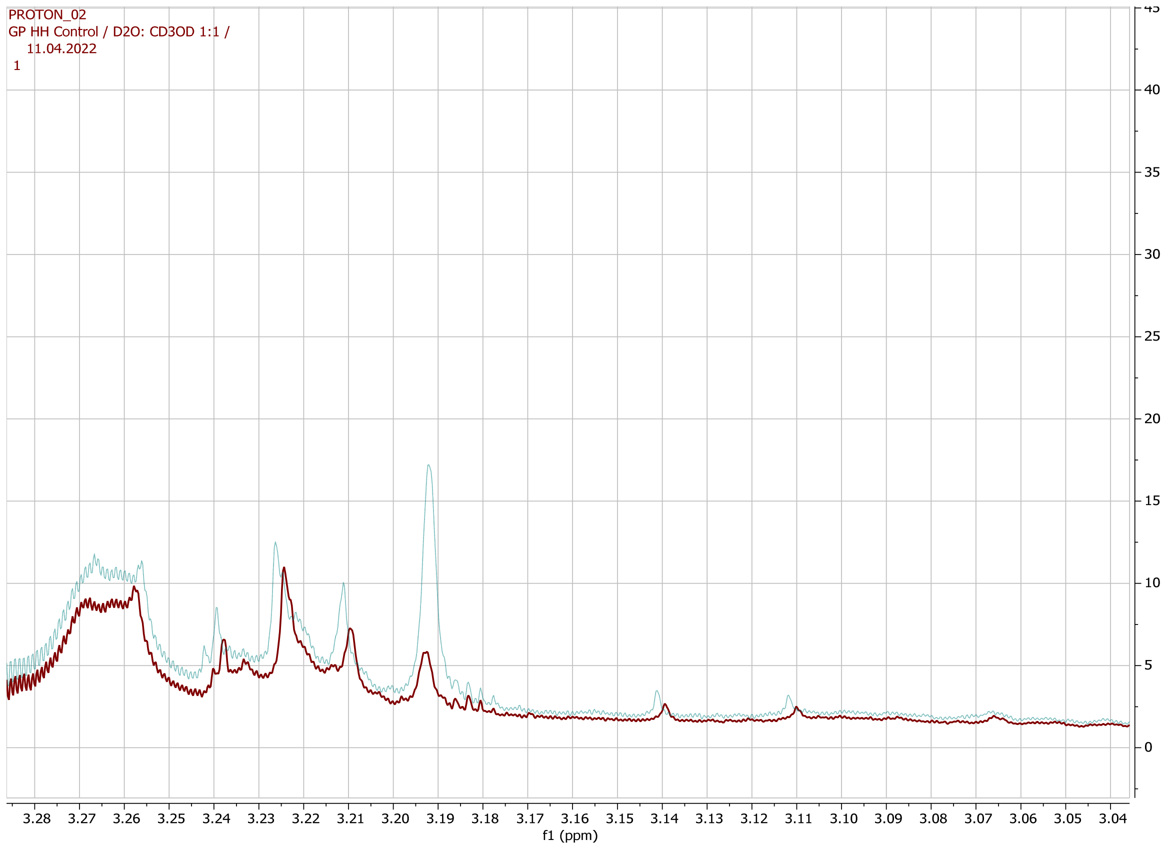

Figure S6. Spiking of the leachate sample with choline. The peaks align at 3.192 ppm.

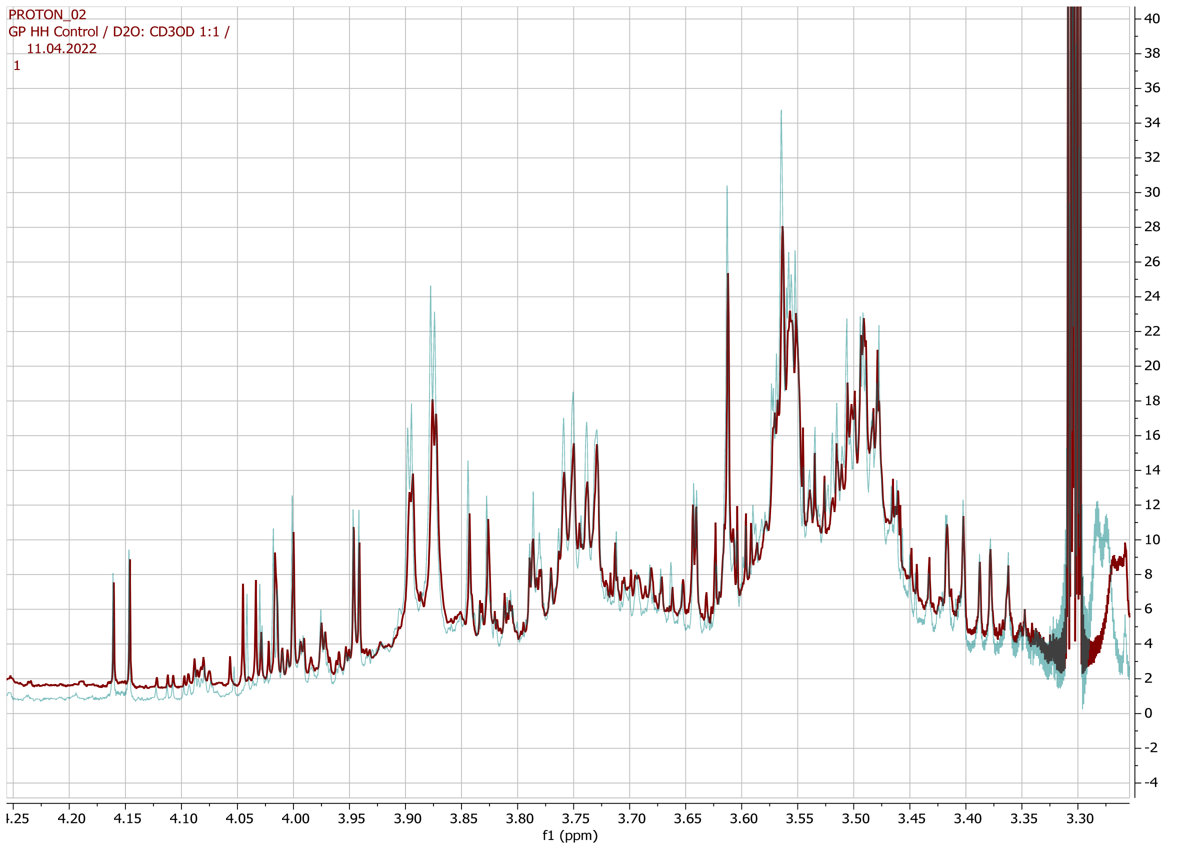

Figure S7. Spiking of the leachate sample (red) with lactose. The various lactose peaks align at 3.28, 3.55, 3.75, 3.85 and 3.95 ppm.

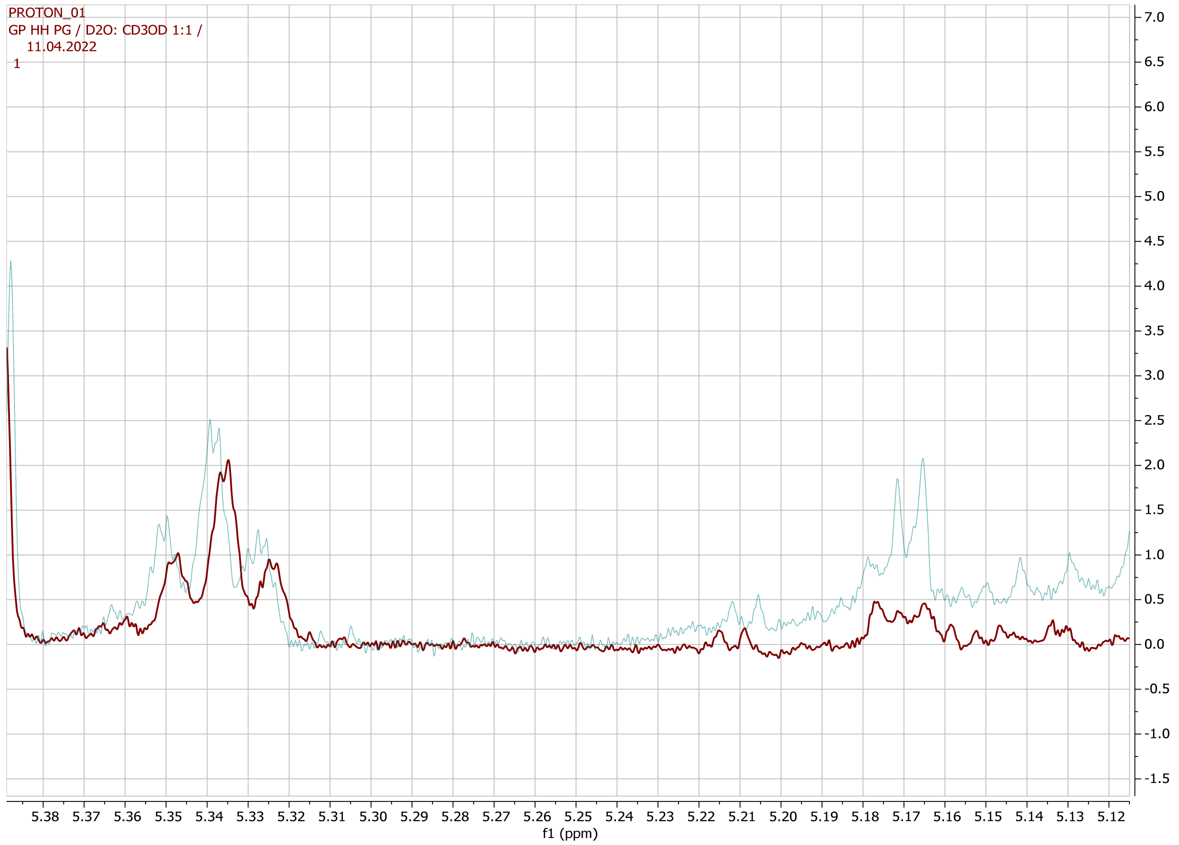

Figure S8. Anomeric proton peak of lactose at 5.17 ppm.

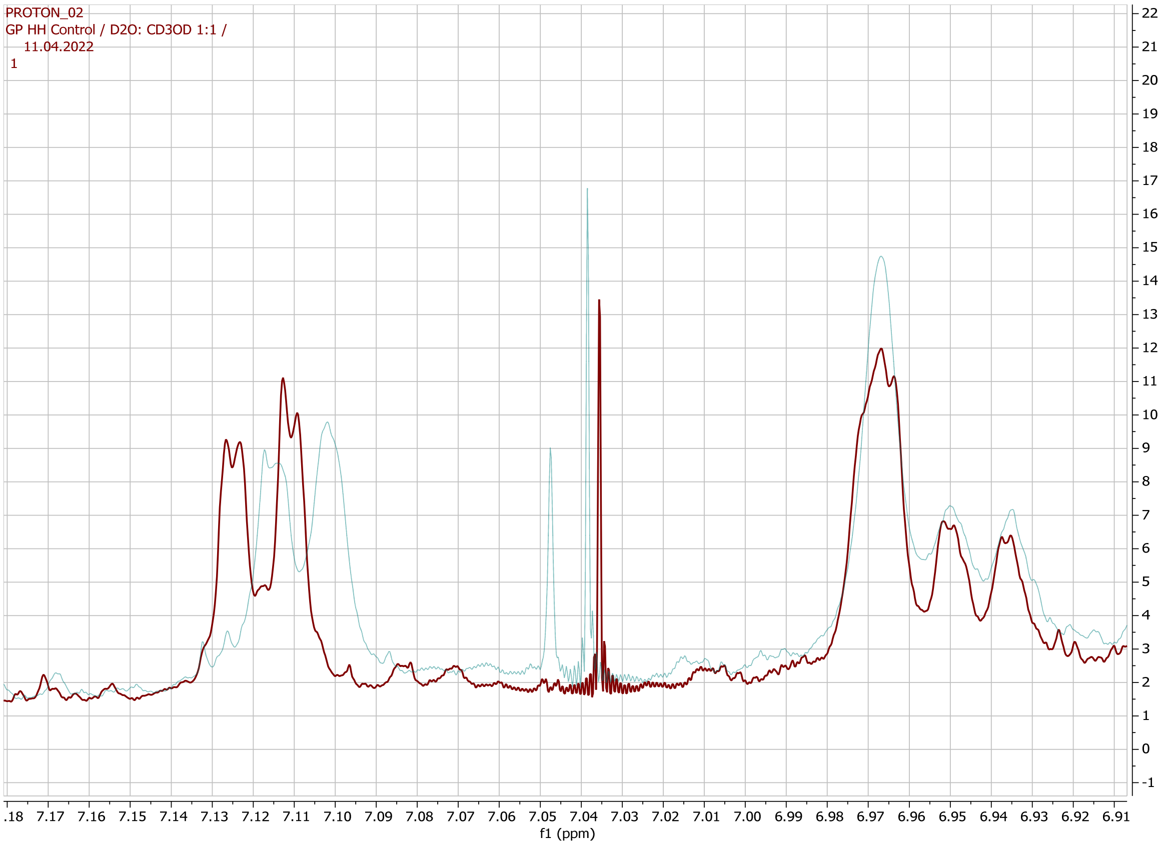

Figure S9. The leachate extract (red) spiked with gallic acid. As the peaks are not aligning, and it cannot be determined if the peak at 7.05 is present in the leachate sample, it could not be positively identified.
